# Hypoxemia burden and atherogenic indices in OSAS: changes after CPAP therapy

**DOI:** 10.3389/fmed.2026.1775054

**Published:** 2026-04-13

**Authors:** Yaşar İncekara, Usame Ömer Osmanoğlu

**Affiliations:** 1 Department of Pulmonary Diseases, Faculty of Medicine, Karamanoglu Mehmetbey University, Karaman, Türkiye; 2 Department of Biostatistics, Faculty of Medicine, Karamanoglu Mehmetbey University, Karaman, Türkiye

**Keywords:** %T90/TST, AIP, CPAP, CRI-I, CRI-II, hypoxaemia burden, lipid profile, OSAS

## Abstract

**Objective:**

To evaluate the association between nocturnal hypoxemia burden—expressed as the percentage of total sleep time spent below 90% oxygen saturation (%T90/TST)—and atherogenic indices, including the atherogenic index of plasma (AIP), Castelli risk index I (CRI-I), and Castelli risk index II (CRI-II), in obstructive sleep apnea syndrome (OSAS); and to assess pre–post changes after adherent continuous positive airway pressure (CPAP) therapy.

**Methods:**

Single-centre retrospective–prospective cohort of adults with moderate–severe OSAS. Hypoxemia burden was quantified as the percentage of total sleep time with peripheral oxygen saturation (SpO₂) < 90% (%T90/TST) and grouped as Low (≤5%), Mild (>5–≤10%), Moderate (>10–≤25%), and Severe (>25%). The lipid panel—total cholesterol (TC), low-density lipoprotein cholesterol (LDL-C), high-density lipoprotein cholesterol (HDL-C), triglycerides (TG)—and atherogenic indices (AIP, CRI-I, CRI-II) were compared across %T90 groups and within patients before and after ≥3 months of adherent CPAP (device-verified ≥4 h/night on ≥70% of nights).

**Results:**

One hundred and four patients were included. Higher %T90 was associated with a progressively more atherogenic profile: LDL-C (*p* = 0.027), TC (*p* < 0.001), TG (*p* < 0.001) and CRI-I (*p* < 0.001), CRI-II (*p* = 0.006), AIP (*p* < 0.001) were significantly higher in the >25% group versus ≤5%. The lowest nocturnal SpO₂ (peripheral oxygen saturation) did not correlate significantly with lipids/indices. After adherent CPAP, LDL-C (118.0 → 102.5 mg/dL), TC (199.0 → 182.0 mg/dL), TG (152.5 → 129.0 mg/dL) and CRI-I (4.37 → 3.94), CRI-II (2.58 → 2.24), AIP (0.55 → 0.46) decreased significantly (all *p* < 0.001), while HDL-C did not change (*p* = 0.570). Primary endpoints (CRI-I/II, AIP) remained significant after Bonferroni correction.

**Conclusion:**

In OSAS, greater hypoxemia burden (%T90/TST) is linked to a more atherogenic lipid profile, and adherent CPAP is associated with a meaningful reduction in atherogenic load (AIP, CRI-I/II). These findings indicate that nocturnal hypoxaemia, captured using index-based measurements (AIP, Castelli I/II) as well as traditional lipids, is associated with a general reduction in atherogenic risk.

## 1 Introduction

Obstructive sleep apnoea syndrome (OSAS) is a condition characterised by partial or complete obstruction of the upper airway during sleep, leading to a decrease in blood oxygen saturation and sympathetic activation. In untreated cases, multiple systems may be affected, including neurocognitive disorders, hypertension, cardiovascular and cerebrovascular diseases, and metabolic dysfunction (1–3).

The nocturnal hypoxaemia burden associated with OSAS is typically assessed using a nightly finger oximeter; the primary measures are the oxygen desaturation index (ODI) and the time spent below 90% saturation (T90) (4). %T90/TST (the ratio of the total sleep time spent below 90% blood oxygen saturation) reflects the total duration of hypoxia throughout the night (5). Hypoxaemia burden is a valuable indicator in risk assessment, easily measured with low-cost oximeters. Indeed, high hypoxic burden and T90 have been associated with higher mortality and hypertension risk in cardiovascular diseases (6–8). The nocturnal hypoxia caused by OSAS is explained by a pathophysiological chain leading to multi-organ/system damage via sympathetic activation, inflammation, oxidative stress, metabolic disorders, and endothelial dysfunction (9). Atherosclerosis is a progressive process characterised by the accumulation of lipid and fibrous elements in large arteries, serving as the fundamental intermediate mechanism in most cardiovascular events (10). Although the underlying mechanisms have not been fully elucidated, the need for reliable biomarkers persists. In this context, atherogenic indices — Castelli Risk Index-I (CRI-I = TC/HDL-C), Castelli Risk Index-II (CRI-II = LDL-C/HDL-C) and Plasma Atherogenic Index (AIP = log10[TG/HDL-C])—are being evaluated as new biomarkers associated with cardiometabolic diseases (11, 12). Large cross-sectional studies have demonstrated an increased prevalence of dyslipidaemia in patients with OSAS (13). Dyslipidaemia is a fundamental component of the pathophysiology of atherosclerosis; furthermore, OSAS may accelerate atherosclerosis associated with dyslipidaemia through pathways such as the oxidation of LDL-C particles (14). An increase in AIP has been associated with cardiovascular disease (CVD) and, in particular, myocardial infarction (MI) risk, a component of coronary heart disease (CHD), in OSAS (15–17).

Multiple mechanisms contribute to dyslipidaemia in OSAS: a tendency towards a high-calorie/high-fat diet, a decrease in postprandial triglyceride clearance due to reduced lipoprotein lipase activity, an increase in hepatic TG/VLDL production due to chronic intermittent hypoxaemia, accelerated release of free fatty acids from adipose tissue due to sympathetic discharges associated with OSAS, and weakened reverse cholesterol transport due to decreased apolipoprotein A synthesis (18). The primary treatment approach for OSAS, continuous positive airway pressure (CPAP), is theoretically expected to correct dyslipidaemia. Indeed, studies reporting positive effects on metabolic syndrome components exist in the literature; however, findings regarding its effects on blood lipid profiles are heterogeneous and do not provide definitive conclusions (19, 20). We believe that changes in AIP and CRI-I/II, along with traditional lipids, may be indicators of the overall atherogenic risk in OSAS and the associated post-CPAP modification.

This study systematically investigates the relationship between %T90/TST-based hypoxaemia burden and lipid panels and atherogenic indices (CRI-I, CRI-II, AIP) in OSAS; it also examines the biochemical changes accompanying the resolution of hypoxaemia following adherent CPAP. It is hoped that the evidence obtained will help to better define the effect of hypoxaemia-focused treatment strategies on atherogenic risk.

## 2 Materials and methods

### 2.1 Study design and ethical approval

This was a single-centre retrospective–prospective cohort study. The retrospective component comprised baseline polysomnography/overnight oximetry variables and pre-CPAP laboratory records collected as part of routine care (data window: 01 January 2022–30 June 2025). The prospective component consisted of a scheduled follow-up visit after ≥3 months of device-verified CPAP use, during which a fasting lipid panel was re-measured. The study adhered to the Declaration of Helsinki and was approved by the Karamanoğlu Mehmetbey University Local Scientific Medical Research Ethics Committee (Approval No. 24–2025/01). For the retrospective part, the use of de-identified data was approved; for the prospective visit, written informed consent was obtained.

### 2.2 CPAP adherence definition

Adherence was defined *a priori* as device-verified ≥4 h/night on ≥70% of nights over a continuous period of ≥90 days (≥3 months). Only patients meeting this predefined adherence threshold were included in the paired pre–post analyses.

Inclusion criteria:

1. Diagnosis of moderate or severe OSAS by polysomnography (PSG) (AHI ≥ 15/h),
2. Initiation of CPAP treatment and regular use for at least 3 months,
3. At least one lipid panel (TC, LDL-C, HDL-C, TG) recorded prior to CPAP treatment,
4. Repeat lipid panel measurement at the follow-up visit (≥3 months).

Exclusion criteria:

- CPAP non-compliance (≥70% of nights with >4 h/night usage in device records),
- Initiation/change in lipid-lowering therapy (statin, fibrate, etc.) between measurements*.
- Causes of secondary dyslipidaemia (nephrotic syndrome, active thyroid disease, Cushing’s syndrome, etc.),
- Acute inflammatory condition/infection, advanced renal/hepatic failure, pregnancy.

### 2.3 Polysomnography and oximetry variables

A 12-channel polysomnographic recording system (Rembrandt, Medcare, Amsterdam, Netherlands) was used to assess sleep, respiratory, and cardiac variables. Scoring was reported by technicians in accordance with AASM criteria. Lowest SpO₂ (%): the lowest oxygen saturation during the night.

Hypoxaemia burden was quantified as the percentage of total sleep time spent with SpO₂ < 90% (%T90/TST). For dose–response assessment and trend testing, %T90/TST was pre-specified into four ordered categories: Low (≤5%), Mild (>5–≤10%), Moderate (>10–≤25%), and Severe (>25%) (7). This ordered stratification enables graded comparisons and p-for-trend analyses and aligns with prior work on hypoxic burden. During in-lab PAP titration, oxygenation improved under CPAP, and—together with device-verified adherence during follow-up—post-treatment nocturnal hypoxaemia was presumed to be substantially reduced. Previous studies have demonstrated that effective CPAP therapy restores upper airway patency in patients with obstructive sleep apnea and markedly reduces intermittent nocturnal hypoxemia, thereby decreasing the cumulative hypoxic burden during sleep. Consequently, indices reflecting hypoxemia burden, such as %T90/TST, are expected to improve with effective CPAP therapy (21–23). However, post-CPAP %T90/TST was not systematically reassessed in routine care and was therefore not a predefined outcome; the primary post-CPAP endpoints were the paired changes in lipid parameters and atherogenic indices after ≥3 months of adherent CPAP.

### 2.4 Biochemical measurements

Venous blood samples were collected in the morning from all patients after at least 8 h of fasting on the morning of the polysomnographic assessment and after at least 3 months of regular CPAP use. TC, LDL-C, HDL-C, and TG were measured using standard laboratory methods with a Beckman Coulter AU5800 autoanalyser. LDL cholesterol levels were calculated using the Friedewald formula, and LDL cholesterol was measured using a homogeneous enzymatic colorimetric method in patients with triglycerides ≥400 mg/dL. Additional variables: CRP, creatinine, leukocytes.

- CRI-I = TC/HDL-C.
- CRI-II = LDL-C/HDL-C.
- AIP = log10(TG/HDL-C).

### 2.5 Statistical analysis

Statistical analyzes were evaluated using the IBM Statistical Package for Social Sciences 25.0 (SPSS, Chicago, IL) program. Correlation heat map graphics were created using the Python 3.7.9 (Delaware, USA) software program. Descriptive statistics are presented as mean±standard deviation and median. Shapiro–Wilk Normality test was applied. Mann–Whitney U Test, Friedman Two-Way Analysis of Variance, and Spearman Correlation Analysis were applied. Mann–Whitney U Test was used to compare the differences according to two independent samples, and Kruskal-Wallis test was used to compare the differences according to four independent samples. Wilcoxon Signed Rank test was used to compare the differences according to two dependent samples. To determine the independent effect of hypoxemia burden on atherogenic indices, multivariable linear regression analyses were performed. Stepwise models were constructed to assess the relationship between %T90/TST (evaluated per 1% increase) and the dependent variables (AIP, CRI-I, CRI-II). Model 1 was unadjusted, and Model 2 was adjusted for BMI. Additional demographic variables, including age, sex, and smoking status, were evaluated but excluded from the final multivariable models as preliminary analyses demonstrated no significant relationship between these factors and the %T90/TST variable. Results are presented as unstandardized (B) and standardized (*β*) coefficients with 95% confidence intervals (CI). Correlation heat map graphics were used to show the correlation between the variables. The interpretation of correlation coefficients was guided by the criteria proposed by Schober et al., 0.00–0.09: no relationship; 0.10–0.39: mild association; 0.40–0.69: moderate association; 0.70–0.89: significant association; >0.90: extremely high association (24). The level of statistical significance was taken as *p* < 0.05.

## 3 Results

A total of 104 adult OSAS patients meeting the inclusion criteria were enrolled (34.6% female). The mean age was 53.7 ± 11.6 years, and the mean BMI was 32.9 ± 5.8 kg/m^2^. Pre-CPAP lipid parameters: total cholesterol 204.5 ± 48.5 mg/dL, LDL-C 122.6 ± 37.4 mg/dL, HDL-C 47.2 ± 11.3 mg/dL, and triglycerides 176.8 ± 87.4 mg/dL. Mean atherogenic indices: CRI-I 4.42 ± 0.88, CRI-II 2.64 ± 0.69, and AIP 0.54 ± 0.24. Inflammatory markers: CRP 3.94 ± 3.59 mg/L and leukocytes 8.33 ± 2.13 × 10^3^/μL. Polysomnography revealed an AHI of 57.2 ± 30.6 events/h, total sleep time of 334.0 ± 69.2 min, and a minimum SpO₂ of 75.2 ± 14.8%. The hypoxaemia burden (T90, the ratio of time spent with SpO₂ < 90% to total sleep time) was 20.0 ± 24.6%; with a categorical distribution of 0–≤5%: 35.6%, 5–≤10%: 12.5%, 10–≤25%: 14.4, and >25%: 37.5%, respectively. The distribution of OSAS severity was moderate: 19.2% and severe: 80.8%. The presence of any comorbidity was 40.4%, and current smoking was 36.5% (Table 1).

**Table 1:** Descriptive characteristics at baseline (Pre-CPAP).

Parameter	Unit	Mean ± SD
Sex		
Female	*n* (%)	36 (34.6)
Male		68 (65.4)
Age	Years	53.67 ± 11.57
HDL-C	mg/dL	47.16 ± 11.25
LDL-C	mg/dL	122.63 ± 37.36
Total cholesterol (TC)	mg/dL	204.47 ± 48.45
Triglycerides (TG)	mg/dL	176.81 ± 87.43
CRI-I (TC/HDL-C)	—	4.42 ± 0.88
CRI-II (LDL-C/HDL-C)	—	2.64 ± 0.69
AIP = log10(TG/HDL-C)	—	0.54 ± 0.06
AHI	events/h	57.23 ± 30.61
T90 (% of TST)	%	19.95 ± 24.55
Lowest SpO₂	%	75.24 ± 14.75
Total sleep time (TST)	Min	334.03 ± 69.22
BMI	kg/m^2^	32.87 ± 5.79
OSAS severity (included strata)		
Moderate	*n* (%)	20 (19.2)
Severe		
Comorbidity (any)		
No	*n* (%)	62 (59.6)
Yes		42 (40.4)
Type 2 diabetes	*n* (%)	8 (7.7)
Hypertension	*n* (%)	24 (23.1)
Coronary artery disease	*n* (%)	10 (9.6)
COPD	*n* (%)	12(11.5)
Smoking		
No	*n* (%)	66 (63.5)
Yes		38 (36.5)
%T90/TST group		
Low (≤5)	*n* (%)	37 (%35.6)
Mild (>5–≤10)		13 (%12.5)
Moderate (>10–≤25)		15 (%14.4)
Severe (>25)		39 (%37.5)

Continuous variables are reported as mean±sd; categorical variables as *n* (%).

Formulas: non-HDL-C = TC − HDL-C; CRI-I = TC/HDL-C; CRI-II = LDL-C/HDL-C; AIP = log10(TG/HDL-C).

T90 (% of TST) = 100 × (time with SpO₂ < 90%)/(total sleep time). T90 categories were defined as 0–≤5%, 5–≤10%, 10- ≤ 25%, > 25% (thus 25.0% → > 25% group).

SD, standard deviation; BMI, body mass index; AHI, apnea–hypopnea index; TST, total sleep time; SpO₂, peripheral oxygen saturation.

This paragraph summarises the baseline (Pre-CPAP) characteristics; matched Pre–Post changes are presented in the next results subheading. There were no significant differences in age and gender distribution among the four groups (*p* = 0.488 and *p* = 0.659, respectively), ensuring that the groups were well-matched for these demographic factors.

As the T90/TST percentage increased, the atherogenic profile deteriorated significantly (Table 2). According to the Kruskal–Wallis test and Dunn post-hoc analyses:

**Table 2:** Lipid panel and atherogenic indices across T90/TST categories.

Parameter	Low (0–≤%5) (*n* = 37)	Mild 5–≤10% (*n* = 13)	Moderate (10–≤25%) (*n* = 15)	Severe (>25%) (*n* = 39)	*p*-value*
	Mean ± SD (median)	Mean ± SD (median)	Mean ± SD (median)	Mean ± SD (median)	
Age (years)	54.30 ± 12.47 (55.00)	51.15 ± 11.70 (48.00)	56.87 ± 10.16 (58.00)	52.69 ± 11.27 (53.00)	0.488
HDL-C (mg/dL)	46.49 ± 9.85 (46.00)	45.31 ± 9.04 (47.00)	49.73 ± 14.10 (47.00)	47.44 ± 12.15 (46.00)	0.895
LDL-C (mg/dL)	107.78 ± 29.09^a^ (107.00)	122.23 ± 30.48^ab^ (129.00)	129.41 ± 44.45^ab^ (123.00)	134.25 ± 39.86^b^ (135.00)	**0.027**
Triglycerid es (TG) (mg/dL)	132.38 ± 43.95^a^ (124.00)	124.92 ± 57.96^a^ (119.00)	187.60 ± 93.74^ab^ (180.00)	232.10 ± 92.55^b^ (212.00)	**<0.001**
Total cholesterol (TC) (mg/dL)	180.22 ± 33.05^a^ (179.00)	192.85 ± 34.89^ab^ (199.00)	215.13 ± 55.38^ab^ (199.00)	227.26 ± 51.15^b^ (220.00)	**<0.001**
CRI-I (TC/HDL-C)	3.94 ± 0.63^a^ (3.89)	4.38 ± 1.11^ab^ (4.15)	4.43 ± 0.96^ab^ (4.58)	4.88 ± 0.73^b^ (4.86)	**<0.001**
CRI-II (LDL-C/HDL-C)	2.35 ± 0.60^a^ (2.27)	2.78 ± 0.91^ab^ (2.60)	2.66 ± 0.82^ab^ (2.53)	2.85 ± 0.55^b^ (2.91)	**0.006**
AIP = log10(TG/HDL-C)	0.44 ± 0.19^a^ (0.44)	0.41 ± 0.25^a^ (0.44)	0.54 ± 0.25^ab^ (0.58)	0.67 ± 0.20^b^ (0.67)	**<0.001**
Ahı (events/h)	41.17 ± 19.80^a^ (40.30)	72.15 ± 35.35^b^ (82.80)	68.49 ± 22.98^b^ (67.70)	63.16 ± 34.14^b^ (57.90)	**0.001**
BMI (kg/m^2^)	29.94 ± 4.10^a^ (30.00)	35.48 ± 5.72^b^ (37.38)	34.22 ± 6.16^ab^ (32.42)	34.25 ± 6.10^b^ (33.60)	**0.001**

SD, standard deviation, *Kruskal–Wallis Test was applied. The superscripts indicate the difference between groups medians. Any measurements without shared superscript letters are significantly different from each other. Bold values indicate statistically significant results (*p* < 0.05).

LDL-C, total cholesterol (TC), and triglycerides (TG) differed significantly across %T90/TST hypoxemia-severity groups—Low (≤5%), Mild (>5–≤10%), Moderate (>10–≤25%), and Severe (>25%) (LDL-C *p* = 0.027; TC *p* < 0.001; TG *p* < 0.001). Levels were highest in the Severe group (>25%) and significantly greater than in the Low group (≤5%), as indicated by post-hoc superscript letters.

Atherogenic indices: CRI-I, CRI-II, and AIP gradually increased as the hypoxaemia load increased (CRI-I *p* < 0.001; CRI-II *p* = 0.006; AIP *p* < 0.001).

CRP levels differed between groups (*p* = 0.025); however, no significant differences were found for HDL-C, white blood cells (WBC), and most biochemical parameters (glucose, creatinine, ALT/AST) (*p* > 0.05) (Table 2).

In multivariable linear regression models, %T90/TST remained an independent predictor of atherogenic risk (Table 3). In the unadjusted model (Model 1), a higher %T90/TST was significantly associated with elevated AIP, CRI-I, and CRI-II (all of *p* < 0.05). This association persisted robustly in Model 2 after adjusting for BMI, demonstrating that the impact of nocturnal hypoxemia on lipid dysregulation is independent of the patient’s body mass. For AIP, the adjusted model yielded a standardized *β* of 0.248 (*p* = 0.017); for CRI-I, *β* = 0.294 (*p* = 0.005); and for CRI-II, *β* = 0.232 (*p* = 0.028).

**Table 3:** Multivariable linear regression models assessing the association between %T90/TST and atherogenic indices.

Dependent variable	Model	Unstandardized B (95% CI)	Standardized *β*	*p*-value*
AIP = log10(TG/HDL-C)	Model 1	0.003 (0.001–0.004)	0.266	0.006
	Model 2	0.002 (0.000–0.004)	0.248	0.017
CRI-I (TC/HDL- C)	Model 1	0.010 (0.003–0.016)	0.270	0.006
	Model 2	0.010 (0.003–0.018)	0.294	0.005
CRI-II (LDL- C/HDL-C)	Model 1	0.005 (0.000–0.011)	0.186	0.049
	Model 2	0.007 (0.001–0.012)	0.232	0.028

Model 1 (Unadjusted), Model 2 (Adjusted with BMI), *Multivariable linear regression analysis was applied.

In the adherent CPAP cohort, a significant improvement was observed in lipid panels and atherogenic indices between pre-CPAP and post-CPAP (Wilcoxon). The change in HDL-C was not significant (46.0 → 45.0 mg/dL; *p* = 0.570). In contrast, LDL-C (118.0 → 102.5 mg/dL; *Δ* = −15.5; %*Δ* = −13.1), total cholesterol (199.0 → 182.0 mg/dL; *Δ* = −17.0; %Δ = −8.5), and triglycerides (152.5 → 129.0 mg/dL; Δ = −23.5; %Δ = −15.4) decreased significantly (all *p* < 0.001). A similar decrease was observed in atherogenic indices: CRI-I (4.37 → 3.94; Δ = −0.43; %Δ = −9.8), CRI-II (2.58 → 2.24; Δ = −0.34; %Δ = −13.2) and AIP (0.55 → 0.46; Δ = −0.09; %Δ = −16.4) decreased in a statistically significant manner (all *p* < 0.001). This pattern supports the notion that correction of hypoxaemia with CPAP is associated with a reduction in atherogenic load (Table 4).

**Table 4:** Changes in lipid panels and atherogenic indices before and after CPAP (adherent cohort, *n* = 104).

Parameter (*n* = 104)	Pre-CPAP	Post-CPAP	*p*-value*
	Mean ± SD (median)	Mean ± SD (median)	
HDL (mg/dL)	47.16 ± 11.25 (46.00)	47.24 ± 9.95 (45.00)	0.570
LDL (mg/dL)	122.63 ± 37.36 (118.00)	103.68 ± 33.70 (102.50)	**<0.001**
Total cholesterol (TC) (mg/dL)	204.47 ± 48.45 (199.00)	179.82 ± 40.79 (182.00)	**<0.001**
Triglycerides (TG) (mg/Dl)	176.81 ± 87.43 (152.52)	143.33 ± 64.11 (129.00)	**<0.001**
CRI-I (TC/HDL- C)	4.42 ± 0.88 (4.37)	3.87 ± 0.80 (3.94)	**<0.001**
CRI-II (LDL-C/HDL-C)	2.64 ± 0.69 (2.58)	2.22 ± 0.65 (2.24)	**<0.001**
AIP = log10(TG/HDL-C)	0.54 ± 0.24 (0.55)	0.45 ± 0.23 (0.46)	**<0.001**

SD, standard deviation, *Wilcoxon Signed Rank test was applied. Bold values indicate statistically significant results (*p* < 0.05).

**Figure 1:**
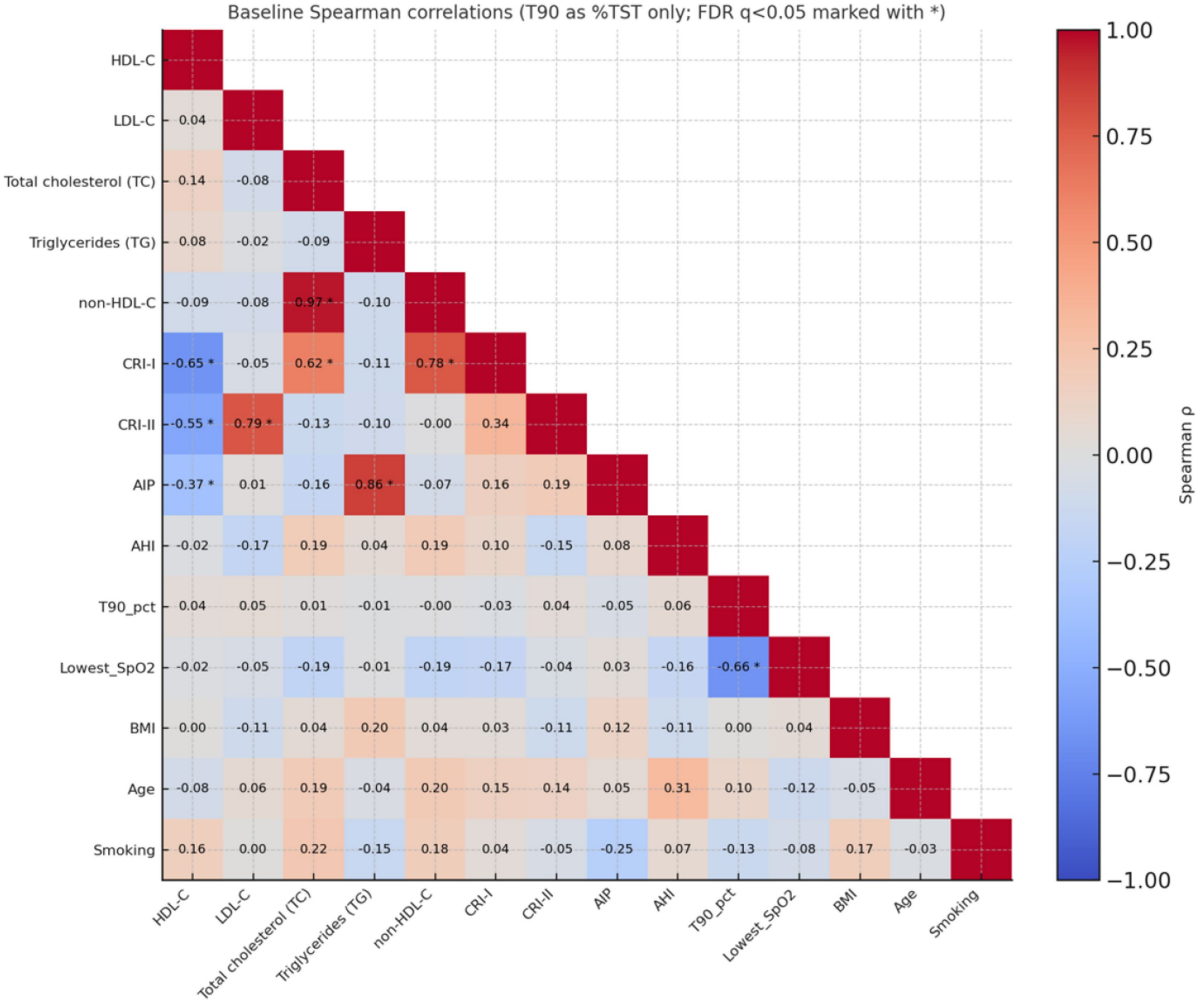
Pre-CPAP Spearman correlation matrix (lipids: HDL-C, LDL-C, TC, TG, non-HDL-C; indices: CRI-I, CRI-II, AIP; sleep measures: AHI, %T90/T, lowest SpO_2_, BMI, age, smoking). Cell *ϱ* values; significance after FDR (q < 0.05) marked with *; lower triangle display, colour scale −1… + 1. Summary trend: %T90/TST↑ → AIP/CRI-I/CRI-II↑; lowest SpO_2_↓.

Spearman correlation analysis (FDR-adjusted, *p* < 0.05) revealed significant relationships in the expected directions between lipid parameters, atherogenic indices, and sleep-related measures. A strong positive relationship was found between LDL-C and total cholesterol (TC), and a very strong positive relationship was found between AIP and triglycerides (TG). CRI-I and CRI-II showed significant positive correlations with LDL-C/TC and negative correlations with HDL-C. T90 (%TST), reflecting the hypoxaemia load, was positively correlated with AIP, CRI-I, and CRI-II and negatively correlated with the lowest SpO₂. As AHI increased, the lowest SpO₂ showed a decreasing trend and clustered in the same direction as the hypoxaemia indicators. There was a very high level of collinearity between T90 (%TST) and T90 (min); therefore, the use of T90 as %TST was preferred in the primary analyses. Overall, as the hypoxaemia burden increased (T90↑), a consistent pattern was observed with deterioration of the atherogenic profile (AIP/CRI-I/CRI-II↑) and worsening oxygenation (rare SpO₂↓) (Figure 1). In multivariable linear regression models, %T90/TST remained significantly associated with AIP (*β* = 0.248, *p* = 0.017) and Castelli Indices (*β* = 0.294, *p* = 0.005 and *β* = 0.232, *p* = 0.028, respectively) after adjusting for BMI, suggesting that the impact of nocturnal hypoxemia on atherogenic risk is independent of obesity levels.

## 4 Discussion

This study shows that a greater nocturnal hypoxemia burden (%T90/TST) is associated with a distinctly more atherogenic lipid profile, whereas correction of hypoxemia with adherent CPAP is accompanied by a meaningful reduction in atherogenic load. Across Low→Severe %T90/TST groups, LDL-C, TC, and TG increased stepwise in parallel with higher AIP and Castelli indices, while the single-point measure of lowest nocturnal SpO₂ was non-informative—supporting %T90/TST as a more relevant metric of cumulative hypoxemia. Quantitatively, adherent CPAP was linked to decreases of roughly 8–15% in LDL-C, TC, and TG, with concordant declines in AIP and CRI-I/II, whereas HDL-C remained stable. Taken together, these findings suggest that index-based assessment can capture overall atherogenic risk in OSA and may aid risk stratification and treatment monitoring, consistent with biological plausibility (intermittent hypoxemia → sympathetic activation, inflammation, oxidative stress, dyslipidemia).

The prevalence of OSAS is high in populations with cardiac comorbidities. Beyond single lipid fractions, composite atherogenic indices may be associated with cardiovascular risk and can improve predictive performance; yet, despite numerous biomarkers studied in OSAS, a clinically actionable panel is not established. Recent work continues to examine the link between OSAS and atherogenic indices (25, 26), and AIP has been reported as a valuable, independent predictor of cardiovascular risk compared with individual lipid parameters (27).

Two main results emerge. First, as hypoxemia burden increased, the lipid profile and atherogenic indices (CRI-I, CRI-II, AIP) worsened progressively. The Severe %T90/TST group (>25%) showed significantly higher LDL-C (*p* = 0.027), TC (*p* < 0.001), TG (*p* < 0.001) and higher indices—CRI-I (*p* < 0.001), CRI-II (*p* = 0.006), AIP (*p* < 0.001)—compared with the Low group (≤5%). No significant correlation was found between the lowest nocturnal SpO₂ and lipids/indices, reinforcing the superiority of cumulative measures (%T90/TST) over single-point readings. Second, following adherent CPAP (≥3 months), LDL-C, TC, and TG decreased significantly, paralleled by significant declines in CRI-I, CRI-II, and AIP; HDL-C did not change. Considered together, conventional lipids and indices converge on the same signal: overall atherogenic risk rises with greater hypoxemia burden and improves after its correction, in line with the notion that composite indices capture risk better than any single lipid alone. Although BMI differed significantly between our hypoxemia severity groups, our multivariable analysis demonstrated that the association between %T90/TST and atherogenic indices persists after controlling for BMI. This finding aligns with the hypothesis that chronic intermittent hypoxemia independently triggers metabolic pathways, such as sympathetic activation and oxidative stress, leading to lipid dysregulation regardless of the patient’s body mass.

Our findings are consistent with a recent meta-analysis showing a reduction in TC with CPAP; importantly, our cohort also demonstrated significant improvements in LDL-C and TG, adding value to the literature (28). Using %T90/TST to quantify hypoxemia revealed a clear dose–response pattern and sharpened group differences on index-based endpoints (CRI-I, CRI-II, AIP). These results align with reports linking OSAS to atherosclerotic plaque burden and atherosclerotic risk (29), and support the utility of %T90/TST as a practical representation of hypoxic load (30). The observed pattern is biologically plausible: chronic intermittent hypoxemia can suppress lipoprotein lipase activity via sympathetic activation, inflammation, and oxidative stress, increase hepatic TG/VLDL production, and weaken reverse cholesterol transport—mechanisms that raise TG and drive higher AIP and Castelli indices (31, 32). Indeed, recent studies have also reported associations between TG and OSA severity/%T90 (32, 33). In our data, AIP correlated positively with %T90/TST and decreased after CPAP (*p* < 0.001), supporting the hypothesis that correcting hypoxemia reduces biochemical atherogenic load.

Similarly, studies have reported elevated CRI-I/CRI-II in OSA (34, 35); in our cohort these indices rose with increasing %T90/TST severity and declined after CPAP. To our knowledge, few studies have jointly evaluated CRI-I/CRI-II and AIP both across %T90/TST-defined severity groups and pre–post CPAP, which may explain the clear and internally consistent pattern we observed. Clinically, %T90/TST is an easily measurable indicator of nocturnal hypoxemia burden that may aid risk stratification, whereas AIP and Castelli indices—simple ratios calculable from routine panels—appear sensitive to the atherogenic pattern of dyslipidemia in OSAS and may complement monitoring of CPAP response.

Limitations: The single-centre design and observational structure limit causal inference. Confounding variables such as diet and physical activity may not have been fully controlled. Although those who changed lipid-lowering medication were excluded from the primary analysis, a residual effect is possible. The lack of association with the lowest SpO₂ may be related to this measure not capturing the hypoxaemia burden as well as T90 and/or power limitations. Nevertheless, adherence verified by device recordings, matched pre–post assessment, T90-based grouping, and index-focused endpoints are strengths of the study.

## 5 Conclusion

Clinical implications: AIP and Castelli indices can be used as actionable surrogate markers of overall atherogenic risk in daily practice. Their sensitivity to adherent CPAP offers potential for index-based risk stratification and monitoring in OSAS. Consequently, as the hypoxaemia burden increases in OSAS, the lipid profile and atherogenic indices deteriorate; following adherent CPAP, the atherogenic burden (AIP, CRI-I/II) decreases. These findings support the effect of targeted treatments for hypoxaemia on biochemical risk markers and suggest that index-based monitoring with %T90/TST may have clinical value.

## Data Availability

The raw data supporting the conclusions of this article will be made available by the authors, without undue reservation.

## Glossary

AASM: American Academy of Sleep Medicine
AHI: Apnea–Hypopnea Index
AIP: Atherogenic Index of Plasma = log10(TG/HDL-C)
ALT/AST: Alanine/Aspartate Aminotransferase
BMI: Body Mass Index
CAD: Coronary Artery Disease
CPAP: Continuous Positive Airway Pressure
CRI-I: Castelli Risk Index-I = TC/HDL-C
CRI-II: Castelli Risk Index-II = LDL-C/HDL-C
CRP: C-Reactive Protein
CVD: Cardiovascular Disease
HDL-C: High-Density Lipoprotein Cholesterol
IQR: Interquartile Range
LDL-C: Low-Density Lipoprotein Cholesterol
LpL: Lipoprotein Lipase
MI: Myocardial Infarction
ODI: Oxygen Desaturation Index
OSA/OSAS: Obstructive Sleep Apnea
PSG: Polysomnography
TC: Total Cholesterol
TG: Triglycerides
TST: Total Sleep Time
%T90/TST: % of TST spent with SpO₂ < 90%
T90: Minutes spent with SpO₂ < 90
WBC: White Blood Cells
